# [Retracted] MicroRNA‑432‑5p inhibits cell migration and invasion by targeting CXCL5 in colorectal cancer

**DOI:** 10.3892/etm.2025.12872

**Published:** 2025-04-22

**Authors:** Man Luo, Zuowei Hu, Yuefeng Kong, Lingyi Li

Exp Ther Medx 21:301, 2021; DOI: 10.3892/etm.2021.9732

Following the publication of the above paper, it was drawn to the Editor's attention by a concerned reader that, regarding the Transwell migration and invasion assay experiments shown in Figs. 2D and 4D, a large number of the panels in this figure showed evidence of overlapping data [specifically, five pairs of data panels in Fig. 2D, and two pairs of data panels (with differing orientations) in Fig. 4D, were found to be overlapping].

Owing to the large number of data duplication events that have been identified in this pair of figures, the Editor of *Experimental and Therapeutic Medicine* has decided that it should be retracted from the Journal on account of a lack of confidence in the presented data. The authors were asked for an explanation to account for these concerns, but the Editorial Office did not receive a reply. The Editor apologizes to the readership for any inconvenience caused.

